# The Evolution of SlyA/RovA Transcription Factors from Repressors to Countersilencers in *Enterobacteriaceae*

**DOI:** 10.1128/mBio.00009-19

**Published:** 2019-03-05

**Authors:** W. Ryan Will, Peter Brzovic, Isolde Le Trong, Ronald E. Stenkamp, Matthew B. Lawrenz, Joyce E. Karlinsey, William W. Navarre, Kara Main-Hester, Virginia L. Miller, Stephen J. Libby, Ferric C. Fang

**Affiliations:** a Department of Laboratory Medicine, University of Washington, Seattle, Washington, USA; b Department of Biochemistry, University of Washington, Seattle, Washington, USA; c Department of Biological Structure, University of Washington, Seattle, Washington, USA; d Department of Microbiology and Immunology and the Center for Predictive Medicine for Biodefense and Emerging Infectious Diseases, University of Louisville School of Medicine, Louisville, Kentucky, USA; e Department of Microbiology, University of Washington, Seattle, Washington, USA; f Department of Microbiology and Immunology, University of North Carolina School of Medicine, Chapel Hill, North Carolina, USA; g Department of Genetics, University of North Carolina School of Medicine, Chapel Hill, North Carolina, USA; University of Illinois at Chicago

**Keywords:** SlyA, countersilencing, evolution, gene regulation, transcription factors

## Abstract

Gene duplication and subsequent evolutionary divergence have allowed conserved proteins to develop unique roles. The MarR family of transcription factors (TFs) has undergone extensive duplication and diversification in bacteria, where they act as environmentally responsive repressors of genes encoding efflux pumps that confer resistance to xenobiotics, including many antimicrobial agents. We have performed structural, functional, and genetic analyses of representative members of the SlyA/RovA lineage of MarR TFs, which retain some ancestral functions, including repression of their own expression and that of divergently transcribed multidrug efflux pumps, as well as allosteric inhibition by aromatic carboxylate compounds. However, SlyA and RovA have acquired the ability to countersilence horizontally acquired genes, which has greatly facilitated the evolution of *Enterobacteriaceae* by horizontal gene transfer. SlyA/RovA TFs in different species have independently evolved novel regulatory circuits to provide the enhanced levels of expression required for their new role. Moreover, in contrast to MarR, SlyA is not responsive to copper. These observations demonstrate the ability of TFs to acquire new functions as a result of evolutionary divergence of both *cis*-regulatory sequences and in *trans* interactions with modulatory ligands.

## Abstract (precis)

Bacteria primarily evolve via horizontal gene transfer, acquiring new traits such as virulence and antibiotic resistance in single transfer events. However, newly acquired genes must be integrated into existing regulatory networks to allow appropriate expression in new hosts. This is accommodated in part by the opposing mechanisms of xenogeneic silencing and countersilencing. An understanding of these mechanisms is necessary to understand the relationship between gene regulation and bacterial evolution. Here we examine the functional evolution of an important lineage of countersilencers belonging to the ancient MarR family of classical transcriptional repressors. We show that although members of the SlyA lineage retain some ancestral features associated with the MarR family, their *cis*-regulatory sequences have evolved significantly to support their new function. Understanding the mechanistic requirements for countersilencing is critical to understanding the pathoadaptation of emerging pathogens and also has practical applications in synthetic biology.

## INTRODUCTION

As organisms adapt to new or changing environments, their regulatory networks must evolve to ensure that individual genes are appropriately expressed in response to environmental signals (1). An important mechanism for the evolution of conserved essential proteins, including transcription factors (TFs), is gene duplication, which allows the subsequent diversification of gene and protein families and the development of new functions (2). More than 50% of bacterial genes are believed to have descended from original duplication events (3–5), providing a broad foundation from which bacteria can evolve complex and adaptive traits.

The MarR family is an ancient family of TFs, predating the divergence of archaea and bacteria (6). It has undergone extensive gene duplication events, with recent estimates suggesting that bacteria encode an average of seven MarR TFs per genome (7). MarR TFs typically function as environmentally responsive repressors of genes encoding efflux pumps that export xenobiotics, including many antimicrobial agents, and are defined by the presence of a winged helix-turn-helix (wHTH) DNA-binding domain (8). The prototypical MarR protein of Escherichia coli represses a single operon, *marRAB*, which encodes a transcriptional activator (MarA) required for the expression of the AcrAB efflux pump, which in turn confers resistance to β-lactams, quinolones, and tetracyclines (9–11). MarR is allosterically regulated by many small molecules, in particular small aromatic carboxylate compounds such as salicylate, which induce a structural change that reduces the affinity of MarR for DNA (10, 12–14) and derepresses the expression of its cognate promoters. A recent study suggests that MarR can also be inhibited by intracellular copper (Cu^2+^), which oxidizes a conserved cysteine residue at position 80, promoting the formation of disulfide bonds between MarR dimers and causing individual dimers to dissociate from DNA (15). Free copper is thought to be liberated from membrane-bound cytoplasmic proteins during envelope stress induced by antimicrobial agents. Dimerization of MarR TFs is required for DNA binding, as it allows these proteins to recognize palindromic sequences via the α4 recognition helix, which makes sequence-specific contacts with the major groove, while the wing makes sequence-independent contacts via the minor groove (16).

A duplication event producing the SlyA lineage of MarR TFs most likely resulted from an ancient horizontal gene transfer event or from intragenomic recombination of a MarR family TF prior to the divergence of the *Enterobacteriaceae*. SlyA has been best characterized in Salmonella enterica serovar Typhimurium, where it serves primarily to upregulate virulence genes (17–19). Although this contrasts with the classical repressive role of MarR TFs, work in our and other laboratories has demonstrated that SlyA positively regulates genes by a countersilencing mechanism, in which repression of AT-rich promoters by the histone-like nucleoid-associated protein H-NS is relieved by SlyA (17, 20). SlyA cooperatively remodels the H-NS–DNA complex in concert with the response regulator PhoP (20, 21), which is activated by conditions found within phagosomal compartments, including low Mg^2+^ (22), acidic pH (23), and cationic antimicrobial peptides (24). SlyA orthologs, represented by Hor, Rap, and RovA in *Pectobacterium* (*Erwinia*), *Serratia*, and *Yersinia*, respectively (25), are conserved in nearly every species of *Enterobacteriaceae*, even including endosymbionts such as Sodalis glossinidius (26), which have undergone extensive gene loss and degenerative evolution (27). This high degree of conservation suggests that the SlyA lineage occupies an essential role in the regulatory network organization of *Enterobacteriaceae*. Although conclusive mechanistic evidence to demonstrate that other SlyA orthologs function as countersilencers has not yet been obtained, existing evidence is strongly suggestive of countersilencing, as several are known to upregulate horizontally acquired traits, which are generally repressed by H-NS, in a number of species, including *Yersinia* spp. (28–30), Dickeya dadantii (31), Pectobacterium carotovorum (25), and Shigella flexneri (32).

TFs can evolve in two ways: in *cis,* through their promoters and associated regulatory elements, both transcriptional and posttranscriptional, altering expression patterns to respond to different environmental and physiological stimuli, and in *trans*, affecting their interactions with cognate binding sites, other proteins, and regulatory ligands. We sought to understand the evolutionary transition of the SlyA/RovA TF lineage from the ancestral function of MarR family TFs as environmentally responsive and dedicated repressors of small regulons to countersilencers of extensive networks of horizontally acquired genes, with a particular focus on in *cis* changes in gene expression and in *trans* changes in modulation by inhibitory ligands. Structural and comparative analyses of representative members of the SlyA lineage were performed to identify the evolutionary changes that allowed SlyA to adopt its new role. Here we show that SlyA has retained an ability to undergo conformational changes in response to aromatic carboxylates, regulate gene expression in an environmentally responsive manner, and repress the expression of a linked drug efflux system. However, SlyA/RovA lineage genes have undergone extensive evolution in *cis* to support the higher levels of expression that are required for countersilencing. Finally, we show that linked efflux pumps are not conserved in some *Enterobacteriaceae,* even though SlyA/RovA TFs have been evolutionarily retained, suggesting that these regulators have been conserved not due to their primordial role in regulating antimicrobial resistance but rather as a consequence of their countersilencing function, which is essential to maintain the regulated expression of horizontally-acquired genes in *Enterobacteriaceae*.

## RESULTS

### Salicylate-mediated inhibition of SlyA activity.

**FIG 1:**
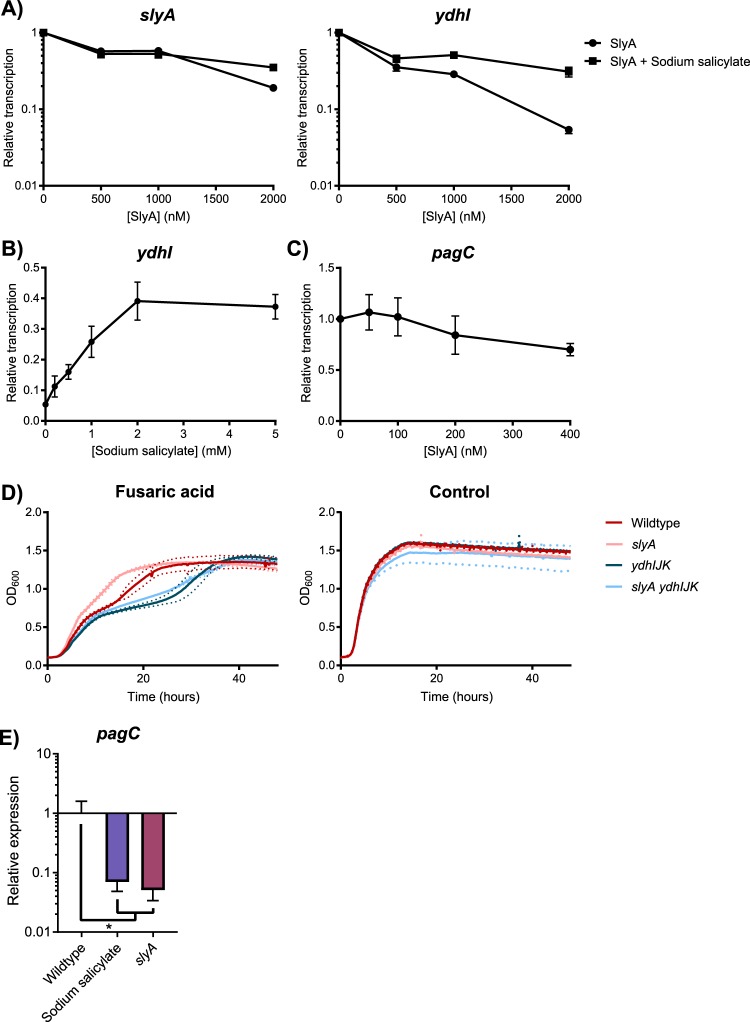
SlyA retains the ancestral functions of MarR family TFs. To determine whether SlyA is an autorepressor like other members of the MarR family, *in vitro* transcription (IVT) assays were performed on supercoiled template DNA containing *slyA* and the divergently expressed *ydhI* gene in the presence of increasing SlyA concentrations (A). Reactions were performed with or without 2 mM sodium salicylate to determine whether SlyA is inhibited by small aromatic carboxylate compounds like other MarR TFs. To determine whether SlyA exhibits a dose-dependent response to salicylate, IVT assays were performed on *ydhI* in the presence of 2 µM SlyA and increasing concentrations of sodium salicylate (B). To confirm that SlyA does not function as an activator at upregulated targets, IVT assays of the model SlyA target gene, *pagC*, were performed in the presence of increasing concentrations of SlyA (C). All IVT data represent the mean ± SEM (*n* ≥ 3). Wild-type, *slyA*, *ydhIJK*, and *slyA ydhIJK* cultures were grown in the presence of 30 µg/ml fusaric acid, and cell density (OD_600_) was measured over time to determine if *ydhIJK* encodes a functional antimicrobial efflux pump (D). Data represent the mean (solid line) from three independent experiments, each consisting of three replicates. Dashed lines represent the SD. The control plot represents the growth curve of each strain in the absence of fusaric acid. To determine whether salicylate also inhibits SlyA countersilencing activity, *pagC* transcripts were quantified by qRT-PCR from early-stationary-phase (OD_600_ ≈ 2.0) cultures in minimal N-medium containing 10 µM MgCl_2_ in the presence or absence of 2 mM sodium salicylate (E). Transcript levels are normalized to *rpoD*, and data represent the mean ± SD (*n* ≥ 3). Asterisk indicates *P* = 0.05.

As environmentally-responsive repressors whose conformation and regulatory actions are modulated by small aromatic carboxylates (12, 14), MarR family TFs are inhibited by salicylate *in vitro* (12). In their structural analyses of SlyA-DNA interactions, Dolan et al. (33) inferred from our structural data (see below) that salicylate might regulate SlyA. Using electrophoretic mobility shift assays, they demonstrated that salicylate inhibits DNA binding by SlyA. To confirm that this influences the function of SlyA as a transcriptional regulator, we performed *in vitro* transcription assays (IVT assays) of *slyA* and the divergently transcribed *ydhIJK* efflux pump operon. Supercoiled plasmid DNA containing the *slyA-ydhIJK* region was incubated with RNA polymerase (RNAP) and increasing SlyA concentrations in the presence or absence of salicylate. SlyA repressed *slyA* transcription approximately 5.3-fold, while *ydhI* transcription was inhibited ∼19-fold (Fig. 1A and B). The addition of 2 mM sodium salicylate reduced SlyA-mediated repression to 2.8-fold and 3.2-fold, respectively, indicating that the sensitivity to aromatic carboxylates observed in classical MarR TFs has been retained by SlyA. IVT assays of the SlyA-upregulated gene *pagC* confirmed that SlyA does not function as a classical activator (Fig. 1C).

We then confirmed that the *ydhIJK* operon encodes a functional antimicrobial efflux pump by growing wild-type and *slyA*, *ydhIJK*, and *slyA ydhIJK* mutant strains in the presence of an aromatic carboxylate with antimicrobial activity, fusaric acid (Fig. 1D). Both the *ydhIJK* and *slyA ydhIJK* mutant strains exhibited delayed growth in the presence of fusaric acid relative to the wild-type strain, suggesting that *ydhIJK* confers fusaric acid resistance in wild-type *S.* Typhimurium. Conversely, *slyA* mutants exhibit improved growth in the presence of fusaric acid, as predicted when *ydhIJK* is derepressed. Collectively, these observations indicate that the *S.* Typhimurium SlyA TF has retained ancestral functions characteristic of the MarR family.

To determine whether salicylate inhibits SlyA-mediated countersilencing as well as repression, expression of the countersilenced *pagC* gene was measured by qRT-PCR in the presence or absence of 2 mM sodium salicylate (Fig. 1E). Wild-type cultures grown in salicylate phenocopied a *slyA* mutant strain, with a >14-fold reduction in *pagC* expression, indicating that salicylate is a general allosteric inhibitor of SlyA function, most likely inducing a structural change that reduces affinity for DNA as described in other MarR TFs (8, 10). Unexpectedly, SlyA retained an ability to interact with DNA upstream of the *ydhI* promoter even in the presence of salicylate (see Fig. S1 in the supplemental material), but this interaction may represent nonspecific interactions with the wing domain, as salicylate inhibited SlyA interaction with a 12-bp DNA region that is highly homologous (75% identity) to the consensus high-affinity binding site (19, 34, 35), centered near the −35 position of the *ydhI* transcription start site (TSS).

10.1128/mBio.00009-19.1 FIG S1 Allosteric inhibition of the SlyA-DNA interaction by small aromatic carboxylates. To determine if small aromatic carboxylates such as salicylate inhibit DNA binding by SlyA, *in vitro* DNase I footprinting was performed on the *ydhI* promoter region in the presence of 2 µM SlyA, in the absence (A) and presence (B) of 2 mM sodium salicylate. Results are presented as a differential DNA footprint analysis (DDFA) plot, representing the difference in fluorescence in relative fluorescence units (RFU) between SlyA-bound DNA and an unbound DNA control (A). Values on the horizontal axis indicate the relative distance from the *ydhI* transcriptional start site (TSS). Downward peaks indicate sites of protection from DNase I digestion, and upward peaks indicate regions of increased DNase I sensitivity, suggesting bending or distortion of the DNA. A DDFA plot representing the difference in RFU between SlyA-bound DNA in the presence and in the absence of salicylate is shown to identify the effect of salicylate on DNA binding (B). There are few significant differences except for a destabilization of the SlyA-DNA interaction between bases −30 and −32 in the presence of salicylate. This indicates that SlyA is still able to interact with DNA in the presence of salicylate, but SlyA binding near positions −30 to −32 is destabilized and likely to be critical for SlyA-mediated repression of *ydhI*. Sequence analysis reveals a region with 75% homology to the high-affinity consensus SlyA binding site (5′-TTAGCAAGCTAA-3′) between bases −28 and −40 (5′-TTGGTAAGCAAA-3′) (19, 34, 35). This region is highlighted in pink in each panel. Data represent the mean ± SD (*n* = 3 independent replicates). Benzoate was used to determine the affinity of the SlyA-carboxylate interaction to avoid inner filter effects associated with salicylate. To confirm that benzoate induces similar conformational changes in SlyA as those observed with salicylate, ^1^H,^15^N-TROSY NMR spectroscopy was performed on 0.3 mM uniformly labeled ^15^N-SlyA in the absence (black) or presence (blue) of 0.5 mM benzoate (C). SlyA in the presence of 2 mM salicylate (red) is overlaid for comparison. To determine the affinity of the SlyA-benzoate interaction, the change in maximum intensity of intrinsic tryptophan fluorescence (*l*_ex_ = 290 nm) of SlyA was measured in relation to increasing concentrations of benzoate (D). The data were fitted to a standard hyperbolic binding isotherm, yielding a *K_D_* of ∼40 µM. Data represent the mean ± SD (*n* = 2). Download FIG S1, TIF file, 0.6 MB. Copyright © 2019 Will et al. 2019 Will et al. This content is distributed under the terms of the Creative Commons Attribution 4.0 International license.

**FIG 2:**
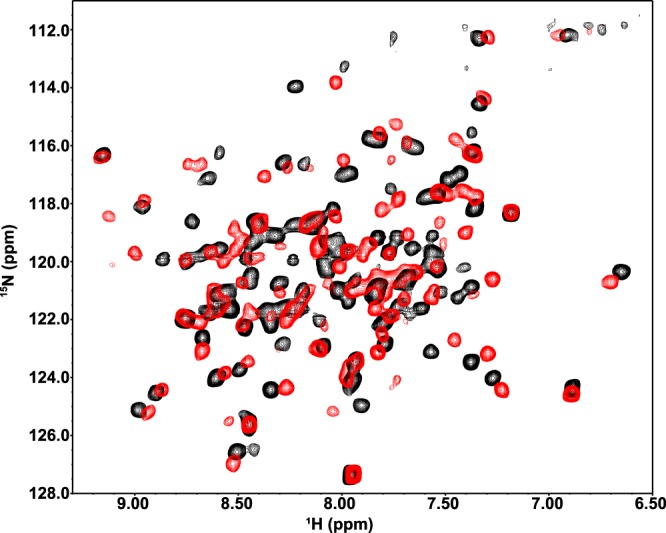
SlyA undergoes significant structural alterations upon salicylate binding. To assess salicylate-induced SlyA structural changes, ^1^H,^15^N-TROSY NMR spectroscopy was performed on 0.3 mM uniformly labeled ^15^N-SlyA in the absence (black) or presence (red) of 2 mM sodium salicylate. (Results of differential DNA footprinting analysis [DDFA] of the SlyA-DNA interaction and ligand binding analyses are shown in Fig. S1.)

To determine whether salicylate-mediated inhibition correlates with structural changes in SlyA, ^1^H,^15^N transverse relaxation-optimized spectroscopy (TROSY) NMR spectra of *S.* Typhimurium SlyA in the presence or absence of salicylate were collected. The apo-SlyA spectrum is well dispersed (Fig. 2), with ∼85% of the expected resonances observed. However, wide variation among individual resonances with respect to peak width and intensity may signify (i) weak nonspecific interactions between SlyA dimers, (ii) various rates of exchange of amide protons with solvent, or (iii) conformational exchange. Previous studies of MarR found that apo-MarR is also highly disordered (8), suggesting that the observed spectral characteristics of apo-SlyA can be ascribed to the presence of multiple conformational states in solution. Addition of salicylate to SlyA induces chemical shift perturbations throughout the spectrum, and nearly 90% of expected resonances are now observed. The large-scale changes in chemical shifts show that backbone amides throughout the protein are stabilized in a different environment relative to apo-SlyA. These observations are consistent with ligand binding to SlyA dimers inducing global structural changes, likely stabilizing a single protein conformation in solution, a conformation that is no longer able to interact with specific high-affinity DNA binding sites. The affinity of the SlyA-ligand interaction was determined via the quenching of intrinsic tryptophan fluorescence in the presence of increasing concentrations of benzoate, an analogous small aromatic carboxylate compound (Fig. S1). Benzoate induces chemical shift perturbations similar to those observed in response to salicylate but does not cause inner filter effects that interfere with fluorescence measurement, as there is relatively little overlap between the UV spectra of benzoate and SlyA, in contrast to salicylate. Although this assay was not able to differentiate between multiple SlyA-benzoate interactions, the *K_D_* (equilibrium dissociation constant) of the SlyA-benzoate interaction was determined to be ∼40 µM, which is similar to the previously determined affinities for EmrR/MprA and HucR (1 to 10 µM) and significantly stronger than that of the MarR-salicylate interaction (0.5 to 1 mM) (13, 36).

### Crystal structure of salicylate-SlyA.

**FIG 3:**
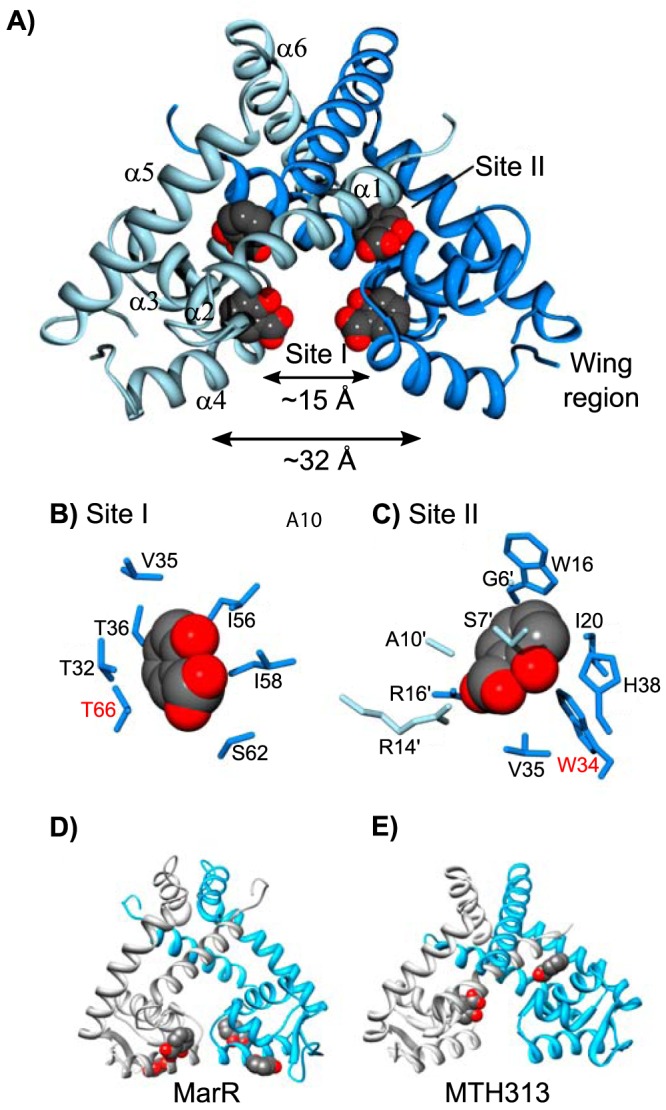
Structure of the SlyA dimer bound to salicylate. The crystal structure of the SlyA dimer with salicylate bound at sites I and II was determined at a resolution of 2.3 Å (A). Residues involved in coordinating bound salicylates are shown for sites I (B) and II (C). Residues required for salicylate-mediated inhibition of SlyA (Fig. 4) are highlighted in red. The structures of salicylate-bound MarR (D) (PDB entry 1JGS) and MTH313 (E) (PDB entry 3BPX) dimers are shown for comparison.

To further analyze the mechanism of allosteric inhibition of SlyA, the structure of the salicylate-SlyA cocrystal was determined. Studies by other groups have previously determined the structure of apo-SlyA and the SlyA bound to DNA (33), demonstrating that SlyA is similar in overall structure to other MarR proteins, consisting of six alpha helices (Fig. 3A). Helices α1, α5, and α6 make up the dimerization domain, while α3 and α4, along with the wing region between α4 and α5, comprise the wHTH DNA-binding domain. These two domains are separated by α2. Dolan et al. (33) previously observed that the two recognition helices of the apo-SlyA dimer are only ∼15 Å apart, in a closed conformation. During interaction with a high-affinity binding site, the helices move a significant distance to accommodate the 32-Å distance between major grooves.

SlyA formed large crystals in the presence of 75 mM sodium salicylate. However, we were unable to obtain usable crystals of apo-SlyA. Diffraction data set and refinement statistics are summarized in Table S1. The two SlyA molecules in the asymmetric unit form two different SlyA dimers in this crystal form. Space group symmetry operations generate the other subunit in each dimer. The dimers are very similar in structure, and further discussion will focus on the dimer formed by polypeptide chain A. We were unable to observe electron density for the tips of the wings, so these regions are absent from our structural model. Difference electron density maps (|Fo|-|Fc|) identified two salicylate molecules bound per SlyA monomer at sites referred to as site I and site II (Fig. 3A to C). Salicylate molecules interact with these sites via hydrophobic interactions with their aromatic rings, while the carboxylate and hydroxyl groups are positioned to interact with the solvent. Site I is composed of residues from α2, α3, and α4, as well as I58 in the loop between α3 and α4, and is well positioned to sterically inhibit DNA binding. It should be noted that the residue numbers in the deposited PDB file are not in register with the residue numbers in this text, which are based on alignment of SlyA orthologs. Comparison with apo-SlyA and SlyA-DNA structures (33) indicates that this salicylate molecule causes the α4 recognition helix to rotate by ∼35° around its axis, disrupting specific contacts with the DNA major groove. Site II is formed by residues from both dimer subunits, almost completely sequestering the salicylate molecule from the solvent. The buried polar groups of salicylate interact with S7′ and R14′ in α1 of one subunit and R17 in α1 and H38 in α2 from the other subunit. A third salicylate binding site was observed on the surface of each subunit of the dimer. However, this site is adjacent to SlyA residues involved in crystal packing contacts and may not be biologically relevant.

10.1128/mBio.00009-19.5 TABLE S1 Crystal structure statistics. Data collection (A) and refinement (B) statistics are shown for the SlyA-salicylate crystal structure. Download Table S1, DOCX file, 0.01 MB. Copyright © 2019 Will et al. 2019 Will et al. This content is distributed under the terms of the Creative Commons Attribution 4.0 International license.

### Mutational analysis of allosteric inhibition of SlyA.

**FIG 4:**
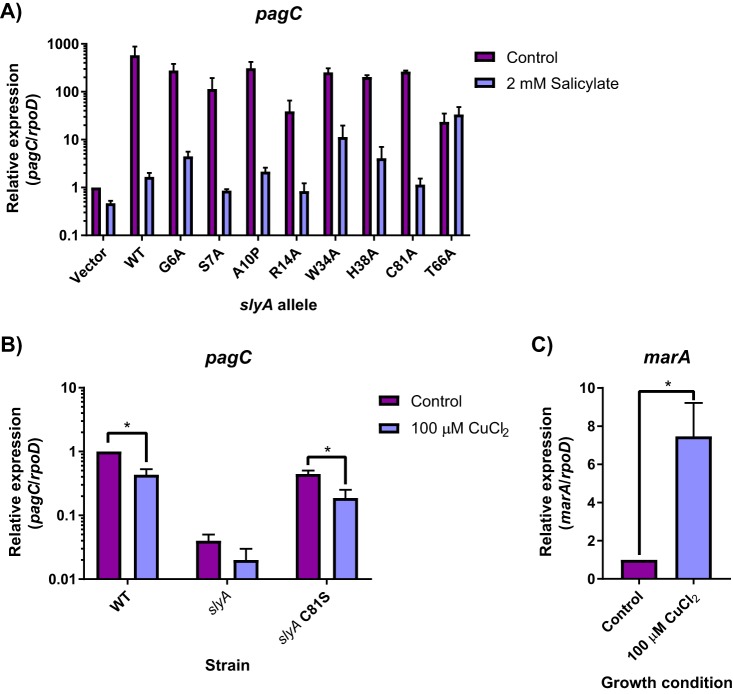
Genetic analyses of allosteric inhibition of SlyA. Selected residues involved in coordinating SlyA-bound salicylate were mutated in pSL2143, a plasmid expressing *slyA* from its native promoter, and assayed for their ability to restore countersilencing in a chromosomal *slyA* mutant. Cultures were induced in 10 µM MgCl_2_-containing medium in the presence or absence of 2 mM sodium salicylate (A). Levels of *pagC* mRNA were quantified by qRT-PCR and normalized to *rpoD*. The mutant strain carrying the empty pWSK29 vector was included as a control. To determine if SlyA was directly inhibited by Cu^2+^ via C81, *pagC* expression was quantified in wild-type or isogenic *slyA* and *slyA* C81S mutant *S.* Typhimurium strains in inducing medium in the presence or absence of 100 µM CuCl_2_ (B). To confirm that Cu^2+^-mediated inhibition occurs under these conditions, *marA* transcription was also quantified in the presence or absence of 100 µM CuCl_2_ (C). Data represent the mean ± SD (*n* = 3). Asterisks indicate *P* < 0.005.

We constructed a series of *slyA* alleles with site-specific mutations of the salicylate-binding pocket in order to test the functional significance of salicylate interactions *in vivo*. When wild-type *slyA* is expressed in *trans* from its native promoter, *pagC* expression increases over 350-fold in inducing medium containing 10 µM MgCl_2_ compared to cultures grown with salicylate (Fig. 4A). We tested 8 different mutant alleles for changes in countersilencing activity in response to salicylate. One allele substituting an alanine for tyrosine 66 in site I (T66A) resulted in complete abrogation of salicylate-mediated inhibition, suggesting that T66 is essential for salicylate binding. However, the T66A mutation also decreased *pagC* expression in the absence of salicylate over 25-fold, indicating that it is required for the wild-type activity of SlyA. A second mutant, W34A in site II, also exhibited reduced salicylate-mediated repression. The analysis of these mutants indicates that both site I and site II bind salicylate *in vivo* and that both sites influence SlyA activity. Notably, both T66 and W34 are absolutely conserved in over 55 enterobacterial genera examined in this study (see below), suggesting that these residues are important for SlyA function throughout the *Enterobacteriaceae* (Table S2).

10.1128/mBio.00009-19.6 TABLE S2 Polymorphisms in salicylate binding sites I and II of SlyA lineage TFs. Download Table S2, DOCX file, 0.01 MB. Copyright © 2019 Will et al. 2019 Will et al. This content is distributed under the terms of the Creative Commons Attribution 4.0 International license.

To determine whether salicylate directly inhibits SlyA *in vivo* or stimulates the release of intracellular Cu^2+^ from membrane-bound proteins to promote disulfide bond formation, we mutated the single cysteine residue in SlyA, C81. SlyA C81A exhibited a similar salicylate inhibition phenotype as wild-type SlyA, indicating that allosteric inhibition does not occur by disulfide bond formation between cysteine residues *in vivo*. We subsequently generated a chromosomal C81S mutant to determine whether SlyA is directly inhibited by Cu^2+^. Transcription of *pagC* in strains carrying wild-type or C81S *slyA* was reduced approximately 2-fold after the addition of 100 µM CuCl_2_ to the growth medium (Fig. 4B). Although this difference was statistically significant (*P* < 0.001), it was also observed in the mutant strain encoding C81S SlyA, indicating that this modest effect is not due to disulfide-dependent tetramerization as proposed for MarR (15). Furthermore, modulation by copper does not appear to be biologically significant in comparison to the 350-fold effect of salicylate. Cu^2+^-mediated derepression of the *marRAB* operon was confirmed under comparable conditions by measuring *marA* expression, which increased approximately 7.5-fold (Fig. 4C).

### Evolutionary analysis of the SlyA TF lineage.

**FIG 5:**
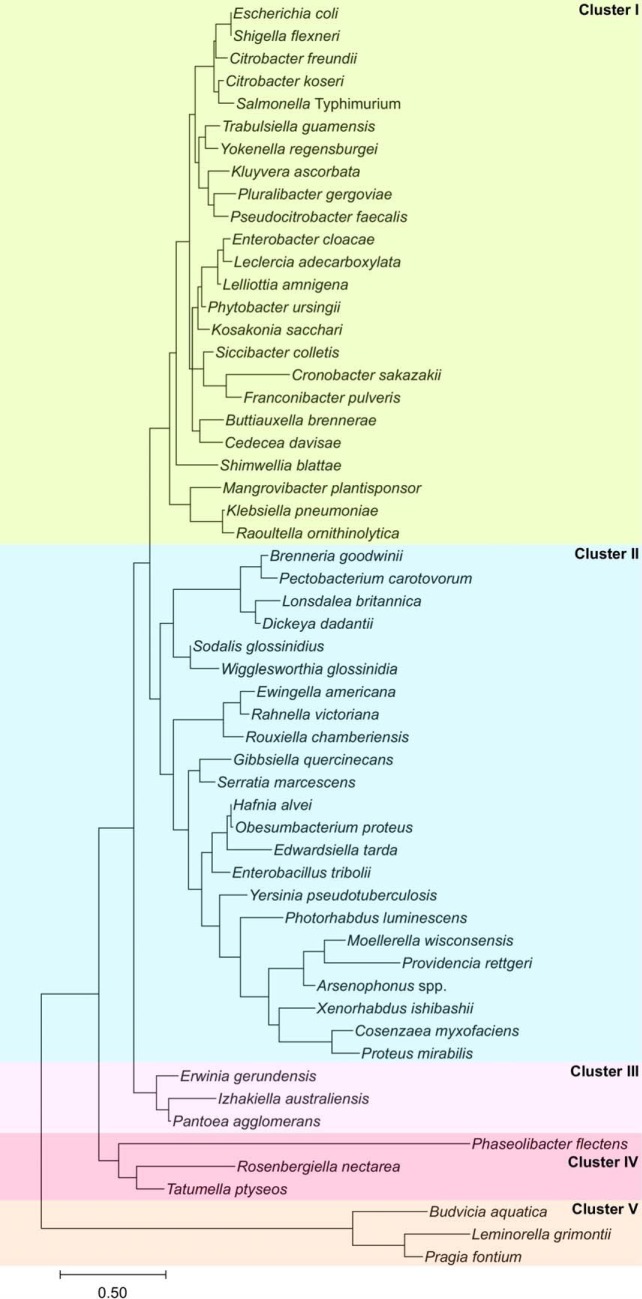
The SlyA TF lineage in *Enterobacteriaceae*. The evolutionary history of the SlyA TF lineage was inferred using the maximum likelihood method and the Le-Gascuel model (89) with Mega X software (90). The tree is drawn to scale with the branch length representing the number of substitutions per site. The *slyA* genes have evolved in five major phylogenetic clusters: cluster I comprises most enteric species, including *S.* Typhimurium and E. coli; cluster II is heterogeneous, containing more distantly related pathogenic species from *Proteus* and *Yersinia*, as well as endosymbionts like *Sodalis*; clusters III and IV contain several plant-associated bacteria, such as Pantoea agglomerans and Phaseolibacter flectens; cluster V is comprised of hydrogen sulfide-producing bacteria. (A multiple sequence alignment of selected SlyA orthologs can be found in Fig. S2.)

To identify other variables that may have contributed to the evolution of the SlyA lineage, we performed an evolutionary analysis of species representing 60 genera of *Enterobacteriaceae*, including *Candidatus* organisms for which genomic data were available. SlyA is strongly conserved, with orthologs found in 55 organisms, suggesting that SlyA plays a central role in enterobacterial regulatory circuitry. Genera lacking identifiable SlyA orthologs include *Buchnera*, *Hamiltonella*, *Samsonia*, *Thorsellia*, and *Plesiomonas*, which are only distantly related to *Salmonella* and *Escherichia* in comparison to other members of the *Enterobacteriaceae*. Notably, we were also unable to identify an *hns* ortholog in *Plesiomonas.* Phylogenetic analysis reveals five clusters of SlyA orthologs (Fig. 5), with the general structure of the phylogram resembling the genomic tree for *Enterobacteriaceae* (37). SlyA orthologs in enteric pathogens including E. coli, *S.* Typhimurium, and S. flexneri form cluster I, while cluster II is composed of a more heterogeneous group of organisms, including plant pathogens such as *D. dadantii*, insect endosymbionts like *S. glossinidius*, and more distantly related pathogens such as Yersinia pseudotuberculosis. SlyA orthologs in clusters I and II are known to function as pleiotropic regulators, despite significant divergence from the other clusters (28, 31). Clusters III and IV are comprised of environmental organisms such as the plant-associated species Pantoea agglomerans and Phaseolibacter flectens, while cluster V contains hydrogen-sulfide producing bacteria such as Pragia fontium. This degree of conservation suggests that SlyA diverged from the greater MarR family of TFs prior to the divergence of *Enterobacteriaceae* from other bacteria. Although not strongly conserved outside *Enterobacteriaceae*, SlyA orthologs can be detected in selected species throughout the *Gammaproteobacteria*, indicating that the lineage is ancient. However, the *slyA* gene typically exhibits an AT content (51% in *S.* Typhimurium) marginally higher than that of the chromosomal average (48% in *S.* Typhimurium), suggesting that it may have been duplicated through an ancient transfer event.

10.1128/mBio.00009-19.2 FIG S2 Multiple alignment of SlyA orthologs in *Enterobacteriaceae.* The protein sequences of SlyA orthologs from representative species of *Enterobacteriaceae* were aligned using T-Coffee (91). Amino acid positions are indicated to the left of each sequence. Download FIG S2, EPS file, 0.4 MB. Copyright © 2019 Will et al. 2019 Will et al. This content is distributed under the terms of the Creative Commons Attribution 4.0 International license.

To determine if allosteric inhibition is likely to be conserved throughout the SlyA lineage, we aligned the sequences of SlyA orthologs from different bacterial species to analyze the conservation of residues involved in salicylate binding (Table S2). Cluster I exhibited nearly complete conservation of the salicylate-binding residues, with only four of 24 genomes encoding polymorphisms. Cluster II exhibited the greatest variation, with 16 of 23 genomes encoding polymorphisms, including 10 which encoded the polymorphism H38Y. We identified only one polymorphism in site I in six genomes, a T32I substitution, suggesting that site I is particularly important for SlyA function. As further evidence of the importance of site I, a T66A mutation abrogated allosteric inhibition of SlyA. The central residues of site II (R14, W16, R17, and W34) are similarly conserved, suggesting that these residues are also important for SlyA function. We were able to mutate two of these residues (Fig. 4A), R14 and W34, and found that W34 is also involved in allosteric inhibition. Individual polymorphisms such as T32I and H38Y appear to have evolved independently in multiple clusters, suggesting purifying selection, although their effect on SlyA activity is currently unknown. Alignment of SlyA protein sequences from representative species of each of the five clusters revealed that most sequence variation occurs in the carboxyl terminus oligomerization domain (Fig. S2), suggesting that ligand sensitivity and DNA binding are conserved features of the SlyA lineage.

**FIG 6:**
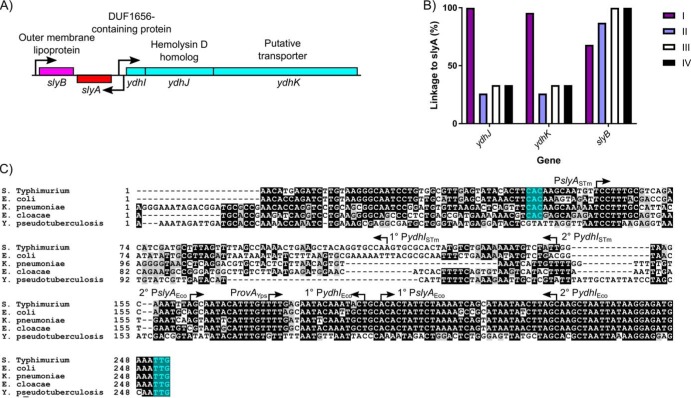
Genetic linkage and *cis*-regulatory evolution of *slyA* in *Enterobacteriaceae*. The *slyA* region of *S.* Typhimurium and closely related enteric species is diagrammed in panel A. Arrows indicate TSSs, and boxes represent protein coding sequences. The *ydhIJK* efflux pump operon is transcribed divergently from *slyA*. A protein of unknown function is encoded by *ydhI,* and *ydhJ* and *ydhK* encode a hemolysin D/EmrA homolog and a transporter protein, respectively. *slyB* encodes a putative outer membrane lipoprotein, transcribed convergently to *slyA*. The linkage of *slyA* to *slyB*, *ydhJ*, and *ydhK* is determined for all of the species from clusters I to IV shown in Fig. 5 (B). Species from cluster V do not exhibit linkage to these genes and are not shown. A multiple sequence alignment of the *slyA* promoter region from the closely related species *S.* Typhimurium, E. coli, K. pneumoniae, and E. cloacae, as well as more distantly related Y. pseudotuberculosis (C). Arrows indicate the position and orientation of previously characterized TSSs (30, 55, 57). Where multiple TSSs have been identified, the primary (1°) and secondary (2°) start sites are indicated. The *slyA* (positions 248 to 250) and *ydhI* (positions 47 to 70, dependent on the species) start codons are highlighted in teal when present. (Alignments of this region from representative species of *Salmonella*, *Escherichia*, and *Yersinia* are shown in Fig. S3. A phylogenetic analysis of the *slyA* promoter region of a subset of genera is shown in Fig. S4.)

As the ancestral function of MarR TFs is the negative regulation of genes encoding drug efflux pumps, a function that is conserved in *S.* Typhimurium, we examined *slyA* orthologs throughout the *Enterobacteriaceae* for linkage to flanking genes of known or hypothetical function: *slyB*, which encodes a putative outer membrane lipoprotein with no observed phenotype; *ydhJ,* which encodes a hemolysin D homolog; and *ydhK*, encoding the efflux pump (Fig. 6A and B). It should be noted that *ydhJ* also exhibits homology to *emrA*, which encodes an antimicrobial efflux pump-associated protein linked to and regulated by another MarR family TF called MprA or EmrR (38, 39). We found that cluster I exhibited the strongest linkage, with 100%, 95%, and 100% linkage to *ydhJ*, *ydhK*, and *slyB,* respectively, while cluster V, containing the hydrogen sulfide-producing bacteria, exhibited no linkage to *slyA* for any of the genes examined. Outside cluster V, *slyB* was strongly associated with *slyA*, exhibiting 76% linkage overall. However, the *ydh* operon was not strongly linked outside cluster I, exhibiting 26%, 33%, and 33% linkage in clusters II, III, and IV, respectively. In contrast, although *marR* was absent from 26 of the enterobacterial species examined, *marR* was linked to *marAB* in every *marR-*carrying species. This suggests that the primary function of MarR is to regulate the *marRAB* operon, and MarR consequently does not play an important role in enterobacterial regulatory circuitry. In contrast, the loss of the ancestral linkage between *slyA* and *ydhJ* outside cluster I suggests that the SlyA lineage has evolved to serve a distinct function.

10.1128/mBio.00009-19.3 FIG S3 Multiple alignment of the *slyA* regulatory regions of *Enterobacteriaceae*. The 250 bp immediately upstream of the *slyA* ortholog coding sequence from representative species of *Salmonella* (A), *Escherichia* (B), and *Yersinia* (C) were aligned using Pro-Coffee (91). The start codons of *slyA* (positions 248 to 250) and *ydhI* (species dependent; positions 47 to 70 when present) are highlighted in teal. Download FIG S3, EPS file, 1.3 MB. Copyright © 2019 Will et al. 2019 Will et al. This content is distributed under the terms of the Creative Commons Attribution 4.0 International license.

10.1128/mBio.00009-19.4 FIG S4 Phylogenetic analyses of the *slyA* promoters of *Enterobacteriaceae.* Species from genera analyzed in Fig. 5 for which annotated genomic data were available on BioCyc (92) were subsequently used in phylogenetic analyses of the 250-bp region immediately upstream of the *slyA* coding sequence, containing the *slyA* promoter. The evolutionary history of the *slyA* promoter region was inferred using the maximum likelihood method and the Tamura-Nei model (93) with the Mega X software (90). The tree is drawn to scale with branch lengths measured in the number of substitutions per site. Branches are highlighted to indicate the corresponding SlyA cluster (Fig. 5). The promoter regions of some members of clusters III and IV are more closely related to members of cluster II than to other members of their own cluster, suggesting that similar promoters may have evolved in parallel in distantly related species. Download FIG S4, EPS file, 0.6 MB. Copyright © 2019 Will et al. 2019 Will et al. This content is distributed under the terms of the Creative Commons Attribution 4.0 International license.

The *ydhIJK* operon does not appear to have been exchanged for another drug efflux system (Fig. 6C). Sequences homologous to the proximal portion of the *ydhI* gene are detectable even in species that have not retained functional coding sequences (e.g., Y. pseudotuberculosis). However, a 100-bp segment of the *ydhI-slyA* intergenic region, beginning approximately 95 bp upstream of the *slyA* start codon, has undergone extensive mutation (Fig. 6C), suggesting in *cis* evolutionary adaptation in the *slyA* lineage of TFs. We examined the regions upstream of *slyA* in several related species of three genera, *Escherichia*, *Salmonella*, and *Yersinia*, to better understand the recent evolution of this region, and observed that the *ydhI-slyA* intergenic regions exhibit considerable divergence between genera and conservation within genera (Fig. S3). A comparison between the *ydhI-slyA* intergenic regions of Escherichia coli and *Salmonella* Typhimurium and those of Klebsiella pneumoniae and Enterobacter cloacae revealed similar degrees of divergence between each genus, suggesting that an ancestral allele can no longer be defined. A phylogenetic analysis of *slyA* upstream regions in a representative subset of the species described above demonstrated extensive variation throughout the *Enterobacteriaceae* (Fig. S4). However, this variation did not always correlate with SlyA coding region clusters. Although the intergenic regions of cluster I are closely related and may have coevolved with their respective coding sequences, the other clusters are more variable, and some intergenic regions may have evolved independently of their coding sequences.

Examination of the *ydhI-rovA* region in *Yersinia* spp. reveals that *rovA* in Y. pseudotuberculosis (Fig. 6C) and Yersinia pestis (Fig. S3) is not linked to a functional *ydhIJK* operon, whereas *ydhIJK* is retained in Yersinia enterocolitica. This indicates that *ydhIJK* was not lost by the former species until after the divergence of *Yersinia* from other *Enterobacteriaceae* and suggests that other species of *Enterobacteriaceae* are also likely to have lost *ydhIJK* recently, as each became adapted to its own specific niche. This represents an example of parallel evolution, with multiple species independently losing *ydhIJK* to accommodate *in cis* evolution as their respective SlyA orthologs adapted to new roles.

### Functional characterization of a distantly related SlyA ortholog.

To understand how the SlyA lineage adapted to its emergent role as an important pleiotropic regulatory protein in the *Enterobacteriaceae*, we compared the functions of SlyA from *S.* Typhimurium (SlyA_STM_) and RovA of Y. pseudotuberculosis and Y. enterocolitica, a relatively divergent ortholog that also functions as a countersilencer (28–30, 40). RovA of Y. pseudotuberculosis exhibits 76% identity with SlyA_STM_ (Fig. S2). RovA is essential for virulence in Y. enterocolitica and Y. pestis (28, 41) and has been suggested to function both as an activator, interacting with RNAP (42), and as a countersilencer, alleviating H-NS-mediated repression (29, 30, 40). Notably, direct activation has not been demonstrated for any other member of the SlyA lineage, and the most direct evidence to suggest that RovA functions as an activator is derived from IVT studies using small linear fragments of DNA as a template (42). We have previously shown that small linear fragments are not necessarily representative of physiological regulatory events in intact cells and can generate spurious results in IVT assays (17). Genetic analysis of the *inv* gene, which is positively regulated by RovA, in an *hns* mutant strain of E. coli also suggested that RovA functions as both a countersilencer and an activator. However, these experiments failed to consider the potential contribution of the H-NS paralog StpA, which is upregulated in *hns* mutants and can provide partial complementation (43, 44), which complicates the interpretation of regulatory studies in an *hns* mutant strain.

**FIG 7:**
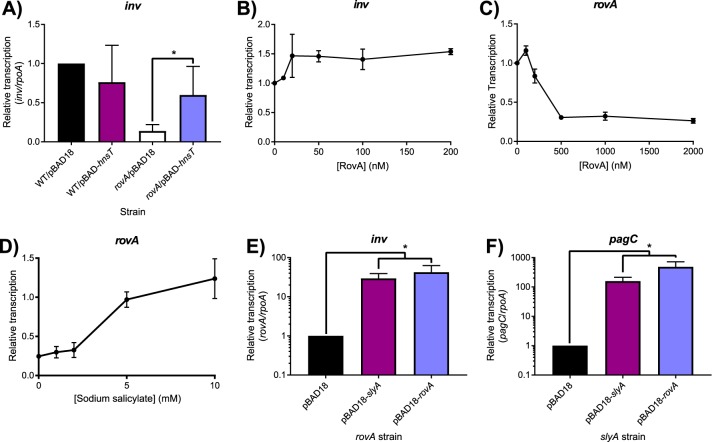
Environmentally responsive repressing and countersilencing functions are conserved in the SlyA TF lineage. To determine whether RovA functions as a countersilencer in Y. pseudotuberculosis, transcription of the Rov-regulated *inv* gene was quantified by qRT-PCR in wild-type or *rovA* mutant strains expressing *hnsT_EPEC_*, which inhibits H-NS (A). Although *inv* expression is decreased in a *rovA* mutant strain, expression is fully restored by the inhibition of H-NS, indicating that RovA functions as a countersilencer. IVT assays of the *inv* regulatory region in the presence of increasing RovA concentrations detected only a minimal impact on transcription levels, suggesting that RovA is unlikely to function as a classical activator (B). IVT assays of *rovA* in the presence of increasing RovA concentrations demonstrate that RovA functions as an autorepressor (C). RovA remains sensitive to inhibition by small aromatic carboxylate molecules, as increasing concentrations of sodium salicylate inhibited RovA-mediated *rovA* repression in IVT assays (D). All IVT data represent the mean ± SEM (*n* ≥ 3). Reciprocal complementation studies were performed by providing either *rovA* or *slyA* in *trans* expressed from the arabinose-inducible pBAD18 vector in *rovA* or *slyA* mutant Y. pseudotuberculosis and *S.* Typhimurium strains, respectively, and measuring transcription of *inv* (E) and *pagC* (F) via qRT-PCR. Transcript levels are normalized to *rpoA* in both species, and data represent the mean ± SD (*n* = 3). Asterisks indicate *P* < 0.05.

We attempted to corroborate the published findings by performing IVT analysis of *inv* expression in the presence of RovA. However, expression of *inv* exhibited only a very modest (∼1.5-fold) increase following the addition of RovA (Fig. 7A), which became saturated at a 20 nM concentration; these results are not supportive of direct *inv* activation by RovA. In contrast, RovA was confirmed to function as an autorepressor, like other MarR family TFs, as IVT analysis demonstrated a 4-fold decrease in *rovA* expression following the addition of RovA protein, with the effect reaching saturation at a 500 nM concentration (Fig. 7B). RovA has also retained the ability to respond to salicylate (Fig. 7C), despite the presence of an H38Y substitution in the second salicylate-binding pocket (Table S2), as 5 mM salicylate completely inhibited RovA-mediated repression, similar to our observations with SlyA. It is also notable that RovA has retained salicylate sensitivity despite the loss of the linked YdhIJK efflux pump in Y. pseudotuberculosis. To prove that RovA functions as a countersilencer in Y. pseudotuberculosis, we measured *inv* expression in wild-type and *rovA* mutant strains expressing H-NST from enteropathogenic E. coli (H-NST_EPEC_). Mutations in *hns* cannot be generated in *Yersinia,* as *Yersinia* spp. carry a single essential *hns* gene, unlike many other members of the *Enterobacteriaceae,* which carry *hns*-like genes such as *stpA* that are able to partially compensate for the loss of *hns*. H-NST_EPEC_ is a truncated *hns* homolog that has been demonstrated to function as a dominant negative form of H-NS by binding and inhibiting the activity of wild-type H-NS protein (45). The expression of *inv* in *rovA* mutant bacteria was approximately 4-fold lower than in wild-type cells (Fig. 7D). However, *inv* expression was fully restored upon inhibition of H-NS by *hnsT*_EPEC_, demonstrating that RovA functions solely as a countersilencer of the *inv* gene. The *rovA* and *slyA* genes are capable of complementing each other in both *S.* Typhimurium and Y. pseudotuberculosis when expressed in *trans* (Fig. 7E and F), upregulating both *inv* and *pagC*, further indicating that RovA functions as a countersilencer, like SlyA. Together, these observations demonstrate that RovA has retained the ancestral characteristic of environmentally-responsive repression exhibited by other members of the MarR TF family, despite being one of the most divergent members of the SlyA lineage. However, in contrast to MarR TFs outside the SlyA lineage, it is also able to function as a countersilencer of horizontally-acquired genes, as exemplified by *inv*.

### SlyA countersilencing requires high expression levels.

**FIG 8:**
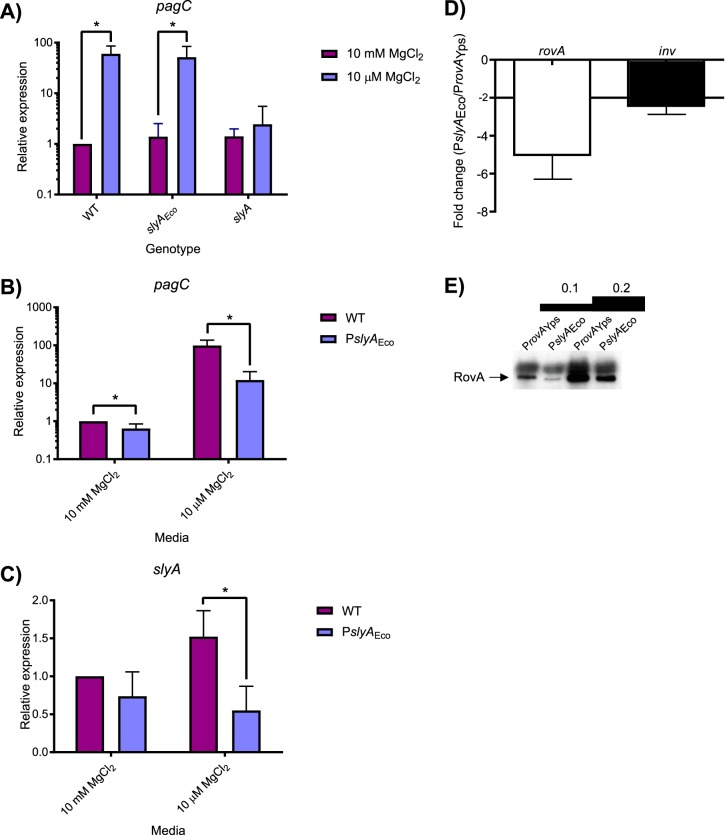
*S.* Typhimurium SlyA is a more effective countersilencer than E. coli SlyA due to higher expression levels. Expression of the SlyA countersilenced *pagC* gene was measured by qRT-PCR in *S.* Typhimurium strains carrying wild-type *slyA*, *slyA_Eco_*, or mutant *slyA* under either inducing (10 µM MgCl_2_) or noninducing (10 mM MgCl_2_) conditions (A). Expression of *pagC* (B) and *slyA* (C) was compared in *S.* Typhimurium strains in which *slyA* transcription was driven by either the wild-type (WT) or E. coli (P*slyA_Eco_*) *slyA* promoter. Transcript levels are normalized to *rpoD*, and data represent the mean ± SD (*n* = 3). Asterisks indicate *P* ≤ 0.05. To compare the strength of the native *rovA* promoter (P*rovA*_Yps_) and P*slyA*_Eco_, the native *rovA* promoter was replaced with the corresponding sequence from E. coli, and the resulting chimera was examined for its ability to regulate the RovA targets *rovA* and *inv* in Y. pseudotuberculosis. Transcripts were quantified by qRT-PCR and normalized to *gyrB* (D). RovA protein levels were confirmed by immunoblot analysis using 0.1 and 0.2 OD_600_ of culture after 6 h of growth (left two and right two lanes, respectively) (E). The position of RovA is indicated by an arrow.

It is interesting that SlyA does not play a major regulatory role in Escherichia coli, the best-studied member of the *Enterobacteriaceae*. SlyA is known to regulate only two E. coli genes, *hlyE* and *fimB* (46–48), despite exhibiting a high degree of homology (91% identity) to SlyA in *S.* Typhimurium. To better understand the different roles of SlyA in the regulatory hierarchy of E. coli and *S.* Typhimurium, we performed a comparative genetic analysis of the *S.* Typhimurium (*slyA*_STm_) and E. coli (*slyA*_Eco_) alleles. In allelic exchange experiments, the *slyA*_STm_ coding sequence was swapped with *slyA*_Eco_ to determine the effect on the expression of the countersilenced *S.* Typhimurium *pagC* gene (Fig. 8A). We observed that SlyA_Eco_ is able to countersilence the expression of *pagC* similarly to SlyA_STm_, suggesting that the diminished role of SlyA in E. coli is not attributable to differences in protein sequence. This suggested that the importance of different SlyA lineage proteins within their respective regulatory networks may be the result of the different *in cis* evolutionary pathways identified in our phylogenetic analysis (Fig. 6 and Fig. S4), resulting in differences in levels of expression. To test this hypothesis, *slyA* intergenic region-ORF chimeras were constructed and assessed for their ability to countersilence *pagC*. To avoid potentially confounding results due to multiple transcriptional start sites (TSSs), we exchanged the intergenic regions beginning immediately upstream of each start codon. Countersilencing was assessed in a *slyA* mutant carrying pKM05, a plasmid with the *slyA*_STM_ ORF transcribed by the E. coli promoter (P*slyA*_Eco_), or pKM07, a plasmid with the *slyA*_STM_ ORF transcribed by its native promoter (P*slyA*_STM_). Although *pagC* expression was similar with either construct under noninducing conditions, expression was approximately 10-fold lower with *slyA* expressed from P*slyA*_Eco_ under inducing conditions (Fig. 8B), suggesting that the diminished role of SlyA in E. coli is at least partially attributable to differences in *slyA* expression levels in *S.* Typhimurium and E. coli. Although *slyA* expression driven by P*slyA*_Eco_ is only slightly lower under noninducing conditions, it is approximately 3-fold greater when driven by P*slyA*_STM_ under inducing conditions (Fig. 8C), suggesting that P*slyA*_Eco_ is less responsive to environmental signals associated with virulence gene expression in *Salmonella* and thus is unable to regulate *slyA* expression in a manner appropriate for countersilencing. Parallel experiments in Y. pseudotuberculosis revealed that *rovA* is also more strongly expressed from its native promoter (P*rovA*_Yps_) than from a chimera expressing *rovA* from P*slyA*_Eco_ (Fig. 8D and E), despite the fact that P*rovA*_Yps_ has diverged much more significantly from both P*slyA*_Eco_ and P*slyA*_STm_ than P*slyA*_Eco_ and P*slyA*_STm_ have diverged from each other (Fig. S4).

## DISCUSSION

The ancient MarR family of transcription factors is represented throughout the bacterial kingdom and in many archaeal species (36). This study examines how the SlyA/RovA lineage of MarR TFs in the *Enterobacteriaceae* has evolved to acquire the novel function of countersilencing. Our observations demonstrate that SlyA proteins have retained some ancestral features, including the environmentally-responsive repression of small molecule efflux systems, while acquiring the ability to act as pleiotropic countersilencers of horizontally-acquired genes. The latter role has been facilitated by the parallel evolution of *cis*-regulatory elements that support higher levels of gene expression.

SlyA, like other MarR family TFs, is subject to allosteric inhibition by small aromatic carboxylate compounds such as salicylate, which bind and stabilize the SlyA dimer in a conformation unfavorable for DNA binding (Fig. 2; also see Fig. S1 in the supplemental material). However, the specific structure and arrangement of the effector binding sites vary significantly among the MarR family. Our structural data indicate that SlyA binds a total of six salicylate molecules per dimer, at least four of which are likely to be biologically relevant (Fig. 3). Although the general structure and architecture of the salicylate-SlyA complex are similar to complexes formed by other MarR TFs, including MarR (8) and MTH313 from Methanobacterium thermoautotrophicum (49), the various TFs differ significantly in their specific interactions with salicylate. MarR binds a total of four salicylate molecules per dimer, whereas MTH313 binds only two (Fig. 3D and E). All four salicylate-binding sites in MarR flank the wHTH domain and are partially exposed to solvent, whereas none of these sites corresponds to the salicylate binding sites of SlyA, potentially accounting for the significant differences in affinity for aromatic carboxylates between the two proteins. MTH313 binds salicylate asymmetrically at sites similar to sites I and II of SlyA, binding only one salicylate molecule per monomer. It is possible that the structural variability in the MarR family with respect to salicylate binding reflects an inherent evolutionary flexibility and ligand promiscuity. The MarR family sensor region may have evolved to interact with a variety of small molecules, and salicylate may simply represent a promiscuous probe for potential interactions. Recent studies have suggested that the true ligand of MarR may be copper, liberated from membrane-associated proteins during oxidative stress induced by xenobiotic agents. Copper reportedly oxidizes a cysteine residue (C80) to promote disulfide bond formation between MarR dimers, resulting in tetramerization and inhibition of DNA binding (15). As almost all characterized MarR TFs have a reactive cysteine residue corresponding to a region near site II of SlyA, we considered that cysteine oxidation, which has also been described in the MarR family TF OhrR (50), might occur in the SlyA lineage as well. However, mutation of C81, the lone cysteine residue in SlyA_STm_, had a negligible effect on SlyA activity in either the presence or absence of salicylate. The addition of copper to the growth medium had a minimal impact on SlyA activity and was unaffected by mutation of C81, indicating that cysteine oxidation is not a general mechanism for the allosteric inhibition of MarR family TFs. Furthermore, the high affinity of SlyA for aromatic carboxylates (Fig. S1) and the conservation of binding pocket residues (Table S2) reported in this study suggest that environmental sensitivity and allosteric inhibition are conserved features of SlyA activity in its new regulatory role.

A phylogenetic analysis of SlyA orthologs in the *Enterobacteriaceae* demonstrates that the SlyA lineage is strongly conserved, even in endosymbiotic species exhibiting significant genome loss (Fig. 5 and Fig. S2). This suggests that SlyA has a central and essential role in the transcriptional regulatory networks of these species. For example, a recent analysis of the endosymbiont *Wigglesworthia glossinidia* found that *slyA* is subject to significant evolutionary constraints (51). This conservation does not appear to be due to its role as a regulator of antimicrobial resistance, as *slyA* does not exhibit significant linkage to the efflux pump operon *ydhIJK*, outside the enteric pathogens in cluster I (Fig. 6). Rather, SlyA proteins appear to function predominantly as pleiotropic countersilencers, facilitating the integration of horizontally-acquired genes, including virulence genes, into existing regulatory networks. A general countersilencing role has been suggested in multiple enterobacterial species including E. coli (19, 48), *Salmonella* (17, 20, 21), and *Shigella* (32) in cluster I and *Yersinia* (31, 52) and *Serratia* and *Pectobacterium* (*Erwinia*) (25) in cluster II. Although similar evidence does not currently exist for the endosymbionts of cluster II, this may simply reflect the limited genetic analyses that have been performed in these species thus far.

To understand the evolutionary adaptations to accommodate countersilencing by the SlyA/RovA lineage, we compared three orthologs, *slyA*_STM_, *slyA*_Eco_, and *rovA*. The *slyA*_STM_ and *rovA* genes are essential for *Salmonella* and *Yersinia* virulence, whereas *slyA*_Eco_ plays a negligible role in E. coli, despite exhibiting far greater similarity to *slyA*_STM_ than *rovA*. These differing roles are attributable to differences in expression, as SlyA_Eco_ is able to function as a countersilencer in *S.* Typhimurium when its expression is driven by the *S.* Typhimurium promoter (Fig. 6). We also observed that *slyA*_Eco_ transcription is significantly reduced in medium with low Mg^2+^ concentrations, a condition associated with the *Salmonella*-containing vacuole (SCV) of macrophages, in which SlyA cooperates with the response regulator PhoP to countersilence virulence genes necessary for intracellular survival (17). This suggests that the diminished role of *slyA*_Eco_ in E. coli may result from its inability to respond to appropriate environmental cues. This is further reinforced by the observation that RovA of *Yersinia* spp. is able to function as a countersilencer in *S.* Typhimurium when expressed in *trans* from an inducible promoter and that the *rovA* promoter of Y. pseudotuberculosis drives *rovA* transcription more strongly than P*slyA*_Eco_ in a chimeric strain (Fig. 8). This suggests that evolution of the SlyA-RovA lineage in *trans*, particularly DNA binding specificity, has played only a minor role since the divergence of the *Enterobacteriaceae*. This is further supported by a comparison of distantly-related SlyA orthologs, which exhibit most sequence divergence in the C-terminal oligomerization domain and not the N-terminal region containing the wHTH (Fig. S2). We conclude that the ability of a given SlyA ortholog to serve as a countersilencer is contingent on its level and pattern of expression, which may be the product of both transcriptional and posttranscriptional activity, as mutations altering TSS location will also alter the 5′ untranslated region of the *slyA* transcript. Notably, another group recently demonstrated a significant expansion of the SlyA regulon in E. coli when *slyA*_Eco_ is overexpressed, with 30 operons exhibiting regulation by SlyA, 24 of which are also repressed by H-NS (34), indicating that SlyA_Eco_ is theoretically capable of functioning as a countersilencer but is not expressed appropriately. In *S.* Typhimurium, the appropriate conditions are those associated with the SCV. However, SlyA orthologs in other species such as the plant pathogens and endosymbionts of cluster II are likely to require expression under different conditions corresponding to their respective environmental niches. The loss of the divergently-transcribed *ydhIJK* operon in Y. pseudotuberculosis and other enteric species may be a consequence of genetic alterations to enhance *rovA* expression as well as the redundancy of drug efflux pumps. An additional possibility is that enhanced *slyA/rovA* expression might result in hyperrepression of *ydhIJK*, which would negate the usefulness of the pump to the cell. Even the plant pathogens such as *D. dadantii* and *P. carotovorum*, which are most likely to encounter small phenolic compounds in the plant environment (53), have failed to retain *ydhIJK*. It is also notable that the *slyA* orthologs in the four species (E. coli, *S.* Typhimurium, Y. enterocolitica, and Y. pseudotuberculosis) in which the transcriptional start sites have been characterized initiate transcription at different positions (30, 54–57), indicating that each species has evolved its *cis*-regulatory circuit independently (Fig. 6).

Previous studies have demonstrated that regulatory evolution can promote adaptation to new niches (58). The SlyA/RovA TF lineage provides a unique example of parallel regulatory evolution to achieve a common functional objective. Throughout the *Enterobacteriaceae*, the associated *ydhIJK* pump genes have been repeatedly lost, yet their regulators have been retained, presumably to facilitate the evolution of an appropriately responsive regulatory circuit to enable countersilencing. This suggests that SlyA/RovA proteins possess intrinsic features that predispose them for a countersilencing role, perhaps the abilities to respond to environmental stimuli and to recognize a variety of AT-rich target DNA sequences. Studies are under way to characterize these features and to determine their contribution to the evolutionary capacity of *Enterobacteriaceae*.

## MATERIALS AND METHODS

### Bacterial strains and general reagents.

All oligonucleotides and plasmids used in this study are described in Table S3 in the supplemental material. Unless otherwise indicated, bacteria were grown in Luria-Bertani (LB) broth with agitation. Salmonella enterica serovar Typhimurium strains were constructed in the ATCC 14028s background and grown at 37°C, unless otherwise indicated. The 14028s *slyA* mutant strain was described previously (17). Yersinia pseudotuberculosis YPIII (59) and YP107 (60) (a gift from P. Dersch, Helmholtz Centre for Infection Research) were used as the wild-type and *rovA* strains, respectively, and grown at 24°C. A *ydhIJK* deletion mutant was constructed using the λ-Red recombinase system (61) and oligonucleotides WNp318 and WNp319. A *slyA ydhIJK* strain was generated by introducing the *slyA*::Cm cassette from 14028s *slyA*::Cm (17) to 14028s *ydhIJK* via P22HTint-mediated transduction. To exchange the wild-type *S.* Typhimurium *slyA* coding sequence with the E. coli allele, *S.* Typhimurium *slyA* was replaced with a *thyA* cassette, via FRUIT (62), using the oligonucleotides STM-slyA-targ-F and STM-slyA-targ-R. The *slyA* coding sequence from E. coli K-12 was then amplified using the primers STM-Eco-slyA-F and STM-Eco-slyA-R, which include 40 and 41 bases, respectively, from the regions flanking the *S.* Typhimurium *slyA* coding sequence. This fragment was electroporated into the *slyA*::*thyA* mutant strain, and the resulting transformants were plated on minimal medium containing trimethoprim, as described for the FRUIT method (62), to select for replacement with the E. coli *slyA* allele.

10.1128/mBio.00009-19.7 TABLE S3 Oligonucleotides and plasmids used in this study. Download Table S3, DOCX file, 0.02 MB. Copyright © 2019 Will et al. 2019 Will et al. This content is distributed under the terms of the Creative Commons Attribution 4.0 International license.

The chromosomal C81S *slyA* mutation (TGC→AGC) was generated by cloning a 1-kb fragment encoding the C81S mutation into the suicide vector pRDH10 (63) using Gibson Assembly (New England Biolabs, Ipswich MA). The Gibson assembly reaction included PCR products generated with primers JKP736/JKP737 and JKP738/JKP739 and genomic *S.* Typhimurium DNA as well as BamHI-digested pRDH10 to generate pJK723. For integration of *slyA* C81S into the chromosome, S17-1Δλpir (64)/pJK723 was mated with *S.* Typhimurium 14028s/pSW172 (65), plated onto LB + 20 µg ml^−1^ chloramphenicol, and incubated at 30°C overnight. Chloramphenicol- and carbenicillin-resistant (Cm^r^ Carb^r^) colonies that were isolated represented a single-crossover event of pJK723 (Cm^r^) plasmid into the *slyA* region of *S.* Typhimurium 14028s (Carb^r^). Selection for the second crossover event to replace wild-type *slyA* with a *slyA* C81S mutation was performed by plating 0.1 ml of an overnight culture of the Cm^r^ Carb^r^ colony onto LB + 5% sucrose plates and incubating at 30°C overnight. Colonies were then streaked onto LB plates at 37°C, and putative 14028s *slyA* C81S colonies were confirmed by DNA sequencing.

To perform allelic exchange analysis of the *rovA* and *slyA*_Eco_ promoters, YPTB007 was generated from Y. pseudotuberculosis IP32953 (66). YPTB007 is a derivative of IP32953 in which the native *rovA* promoter was replaced with the E. coli *slyA* promoter. Briefly, the 691 bp 5′ of the native *rovA* gene were replaced with the 292 bp 5′ of the E. coli *slyA* gene using pSR47s (67) and homologous recombination. Replacement was confirmed by PCR amplification of the intergenic region and DNA sequencing.

### Cloning.

pSL2143 was generated by amplifying the *slyA*_STm_ region, including its native promoter, with primers slyAcomp-F and slyAcomp-R and ligating into pWSK29 (68) digested with EcoRV. The G6A, S7A, A10P, R14A, W34A, H38A, T66A, and C81A mutants were generated with their respective mutagenic primer pairs (Table S3) using the QuikChange XL site-directed mutagenesis kit (Agilent Technologies, Santa Clara, CA) according to the manufacturer’s protocol. A 2,249-bp region containing both *slyA* and *ydhI* was amplified by PCR using the primers SlyAreg-F and SlyAreg-R. The resulting fragment was digested with BamHI and HindIII and ligated into the low-copy-number IVT scaffold vector pRW20 (17) to generate pRW39. An IVT target containing the 2,379-bp *rovA* region from Y. pseudotuberculosis was generated using the primers BamHI-rovA-F and EcoRI-rovA-R. The resulting fragment and pRW20 were both digested with BamHI and EcoRI and ligated together to construct pRW54. An IVT target containing the 2,708-bp *inv* region from Y. pseudotuberculosis was generated via PCR using the primers BamHI-inv-F and EcoRI-inv-R. The resulting fragment and pRW20 were both digested with BamHI and EcoRI and ligated together to construct pRW55. To perform *rovA* and *slyA* complementation studies, both genes were cloned into the arabinose-inducible expression vector pBAD18 (69). The *rovA* gene was amplified using the oligonucleotides EcoRI-rovA-F and KpnI-rovA-R. The *slyA* gene was amplified using the oligonucleotides EcoRI-slyA-F and KpnI-slyA-R. Both fragments were digested with EcoRI and KpnI and ligated into pBAD18, generating pRW58 (*slyA*) and pRW59 (*rovA*). pRW60 was constructed by cloning an N-terminal 6×His-tagged copy of *rovA* into pTRC99a (70). The *rovA* gene was amplified from YPIII genomic DNA by PCR using the oligonucleotides 6HisRovA-F and 6HisRovA-R. The resulting fragment and pTRC99a were digested with NcoI and BamHI and ligated together. An E. coli P*_slyA_*-*S*. Typhimurium *slyA* coding sequence chimera was constructed using overlapping PCR. The *slyA-ydhIJK* intergenic region was amplified from E. coli K-12 genomic DNA using primers KMp178 and KMp206 and from *S.* Typhimurium 14028s using oligonucleotides KMp177 and KMp206. The corresponding *slyA* ORF from *S.* Typhimurium was amplified with primers KMp207 and KMp181. The ORF and promoter segments were amplified along with 40 bp of complementary overlapping sequence. Products from the ORF and promoter reactions were mixed 1:1 and amplified using primers KMp178 (E. coli) or KMp177 (*S.* Typhimurium) and KMp181 to amplify the promoter/ORF chimeras. The resulting fragment and pTH19Kr (71) were digested with BamHI and HindIII and ligated together, generating pKM05 (E. coli promoter) and pKM07 (*S.* Typhimurium promoter). Plasmids were transformed into *S.* Typhimurium 14028s *slyA* for gene expression analysis. The *hnsT* coding sequence was amplified from E2348/69 genomic DNA using the oligonucleotides EcoRI-hnsT-F and HindIII-hnsT-R. Both pBAD18 and the resulting PCR fragment were digested with EcoRI and HindIII, agarose gel-purified, and ligated together to generate pRW57.

### Fusaric acid resistance assays.

Cultures were grown overnight in LB broth at 37°C and then diluted 1:100. Thirty microliters of this dilution was added to 270 µl of LB containing 30 µg/ml freshly-prepared fusaric acid in 100-well BioScreen plates (Growth Curves, Piscataway, NJ). Cultures were grown with continuous maximum shaking at 37°C, and regular OD_600_ measurements were taken on a BioScreen C MBR. Fresh 20-mg/ml stock solutions of fusaric acid were prepared in dimethyl formamide (DMF).

### *In vitro* transcription.

IVT assays were performed essentially as previously described (17) with the following modifications. All SlyA IVT assays were performed at 37°C. All RovA IVT assays were performed at 24°C. Where indicated, sodium salicylate was added to IVT reaction mixtures prior to the addition of SlyA or RovA. All oligonucleotides and templates used in IVT reactions are indicated in Table S4.

10.1128/mBio.00009-19.8 TABLE S4 Components of IVT reactions. Download Table S4, DOCX file, 0.01 MB. Copyright © 2019 Will et al. 2019 Will et al. This content is distributed under the terms of the Creative Commons Attribution 4.0 International license.

### RNA isolation and qRT-PCR.

RNA was purified using TRIzol (Life Technologies, Carlsbad, CA) according to the manufacturer’s protocols. cDNA was generated using the QuantiTect reverse transcription kit (Qiagen, Hilden, Germany) and quantified in a Bio-Rad CFX96 (Bio-Rad, Hercules, CA), using SYBR Green master mix (72).

For the analysis of *pagC* expression under inducing conditions, *S.* Typhimurium cultures were grown to early stationary phase (OD_600_ ≈ 2.0) at 37°C in LB broth and then washed three times in N-minimal medium containing either 10 µM (inducing) or 10 mM (noninducing) MgCl_2_. Cultures were resuspended in the appropriate N-minimal medium with 2 mM sodium salicylate where indicated and incubated for an additional 30 min at 37°C before cells were harvested for RNA purification studies. For the complementation of *slyA* with *slyA* or *rovA* in *trans*, overnight cultures were diluted to 0.05 OD_600_ and grown for 2 h at 24°C before arabinose was added to a final concentration of 0.02% (wt/vol). Cultures were grown for an additional 6 h before harvesting RNA.

Y. pseudotuberculosis strains YPIII and YP107 were diluted from overnight cultures to 0.05 OD_600_ in LB and grown with shaking at 24°C. For the inhibition of H-NS via H-NST_EPEC_ overexpression, and complementation of *rovA* with *rovA* or *slyA* in *trans*, YPIII and YP107 cultures carrying either pBAD18 or pRW57 were grown for 2 h before arabinose was added to a final concentration of 0.02% (wt/vol). Cultures were grown for an additional 6 h before harvesting RNA. For *slyA*/*rovA* complementation studies, rpoAYS primers targeting *rpoA* were used as loading controls, as *rpoA* is sufficiently conserved between the two species as to allow use of the same primers. For *rovA* promoter allelic exchange analysis, Y. pseudotuberculosis IP32953 and YPTB007 (*n* = 3) were grown at 26°C with aeration. After 15 h, bacterial concentrations were determined by spectrophotometry and diluted to 0.1 OD_600_ in fresh medium. Cultures were grown for 6 h at 26°C before cells were harvested for mRNA extraction. *rovA* and *inv* transcript levels were determined by qRT-PCR using Sybr Green and normalized to *gyrB* transcript levels (73, 74).

### NMR analysis of SlyA-salicylate.

SlyA protein for NMR analysis was prepared as an N-terminal 6×His-tagged protein from cells grown in M9 minimal medium supplemented with ^15^N-ammonium chloride (Cambridge Isotope Labs, Tewksbury, MA). SlyA protein expression was induced by the addition of 2 mM IPTG (isopropyl-β-d-thiogalactopyranoside), and the protein was purified to homogeneity using Ni-affinity chromatography as previously described (21), except that samples were dialyzed in 50 mM Tris-HCl, pH 8.0, 150 mM NaCl, 0.1mM EDTA, and 3 mM DTT following purification. Protein and ligand solutions for NMR experiments were prepared in 25 mM sodium phosphate, 150 mM NaCl buffer at pH 7.0 containing 10% D_2_O. NMR spectra were collected on a Bruker DMX 500-Mhz spectrometer (Bruker, Billerica, MA) on samples equilibrated at 35°C and consisting of 250 to 350 µM ^15^N-labeled His-SlyA in the absence or presence of 2 mM sodium salicylate or 0.5 mM sodium benzoate. Spectra were processed using NMR-Pipe (75) and analyzed using NMR-View (76).

### SlyA-salicylate crystallization.

SlyA was overexpressed and purified for crystallization as previously described (21), except that samples were dialyzed in 50 mM Tris-HCl, pH 8.0, 150 mM NaCl, 0.1 mM EDTA, and 3 mM DTT following purification. Cryo I and II sparse matrix crystallization screens (Emerald Biosystems, Bainbridge Island, WA) were used to determine initial conditions for His-SlyA crystal formation by sitting-drop vapor diffusion in 24-well crystal trays. Equal volumes (4 µl) of SlyA and crystallization solutions were mixed before plates were sealed and kept at room temperature. Crystals appeared within 4 to 7 days in 20% PEG 300, 10% glycerol, 0.1 M phosphate-citrate buffer, pH 4.2, 0.2 M ammonium sulfate (condition 14). Subsequent crystal growth was performed using lab-made 20% PEG 400, 10% glycerol, 0.1 M phosphate-citrate buffer, pH 4.2, and 0.2 M ammonium sulfate. Crystallization of SlyA with sodium salicylate was achieved by adding dilutions of a 2 M sodium salicylate aqueous stock solution to the protein-crystallization solution mixture. SlyA-salicylate crystals appeared within 7 to 10 days. A single crystal appeared at a concentration of 75 mM sodium salicylate and grew to a maximum size of 500 µm by 300 µm by 150 µm (Fig. S4). This crystal was used for X-ray diffraction experiments.

The crystal was frozen at 100 K in its crystallization solution for diffraction data collection on GM/CA-CAT beamline 23-ID-D at the Advanced Photon Source. The space group for the crystals is P21212 with two SlyA molecules in the asymmetric unit. The diffraction data were processed with HKL2000 (77). Data set statistics are shown in Table S1A. The crystal structure of the salicylate complex of SlyA was solved using a model of a previously investigated structure of apo-SlyA (unpublished data). That apo-structure was solved using the molecular replacement program MOLREP (78) with a search model generated by applying Swiss-Model (79) and the SlyA amino acid sequence to PDB entry 2FBH (80). The structural model for the salicylate complex was refined using REFMAC-5 (81) in the CCP4 suite (82). *R*_free_ (83) was calculated using 5% of the data in the test set. A high-resolution limit of 2.3 Å was applied for the refinement, consistent with standards appropriate when the structure was solved. This is the resolution at which *R*_merge_ for the data set drops below 0.40. XtalView (84) and Coot (85) were used to examine sigma A weighted |Fo|-|Fc| and 2|Fo|-|Fc| electron density maps (86). MOLSCRIPT (87) and Raster3D (88) were used to produce structural figures for this paper. Table S1B contains refinement statistics for the structure.

### Ligand binding assays.

To estimate the *K_D_* of ligand binding to SlyA, the quenching of intrinsic tryptophan fluorescence in SlyA was measured in the presence of the aromatic carboxylate benzoate. Benzoate was selected for these experiments because (i) ^1^H,^15^N-TROSY NMR spectra show that binding of benzoate induces chemical shift perturbations similar to those observed with other aromatic carboxylates such as salicylate and (ii) inner filter effects during fluorescence measurements can be avoided since there is little overlap in the UV spectra of benzoate and SlyA, and an *l*_ex_ of 290 nm can be used to stimulate intrinsic SlyA tryptophan fluorescence. Titrations were conducted starting with 0.2 µM His-SlyA in 25 mM NaPO_4_, 150 mM NaCl, pH 7.0, at 25°C followed by sequential additions from an identical sample containing 2 mM sodium benzoate. Emission spectra from 320 to 450 nm (*l*_ex_ = 290 nm) were collected from samples ranging in benzoate concentration from 0 to 200 mM, and the change in maximum intensity was plotted as a function of benzoate concentration. The data were fitted to a standard hyperbolic binding isotherm yielding a *K_D_* of ∼40 µM.

### DNase I footprinting and DDFA.

DNase I footprinting was performed using methods described previously (1). SlyA was incubated at a final concentration of 2 µM in the presence or absence of sodium salicylate with the target plasmid pRW39 for 10 min at 37°C. DNase I was added and allowed to digest the SlyA-DNA complex for 2 min at 37°C before the reaction mixture was quenched with cold stop buffer, purified, amplified by fluorescent primer extension using oligonucleotide 6-FAM-ydhI-R, and analyzed by differential DNA footprinting analysis (DDFA).

### 6×His-RovA purification.

RovA was purified using the same protocol as described previously for SlyA (17). However, overexpression cultures were grown at 24°C to an OD_600_ of 0.5. IPTG was added to a final concentration of 1 mM, cultures were incubated at 24°C for an additional 4 h before the cells were harvested by centrifugation, and cell pellets were stored at −80°C for the subsequent purification of RovA.

### Western blot analysis.

For Western blot analyses, 1.0 OD_600_ equivalent of total bacteria was harvested and lysed in SDS protein loading buffer. Equivalents of 0.1 or 0.2 OD_600_ were separated by SDS-PAGE and transferred to PVDF, and Western blotting was performed with rabbit anti-RovA antibody (1:1,000) (40).

### Data availability.

Coordinates and structure factors have been deposited in the Protein Data Bank with identifier 3DEU.
