# Supplementary figures and images for: The Evolution of SlyA/RovA Transcription Factors from Repressors to Countersilencers in Enterobacteriaceae
Source: mBio. 2019 Mar 5;10(2):e00009-19. doi: 10.1128/mBio.00009-19 (PMC6401476; doi:10.1128/mBio.00009-19)

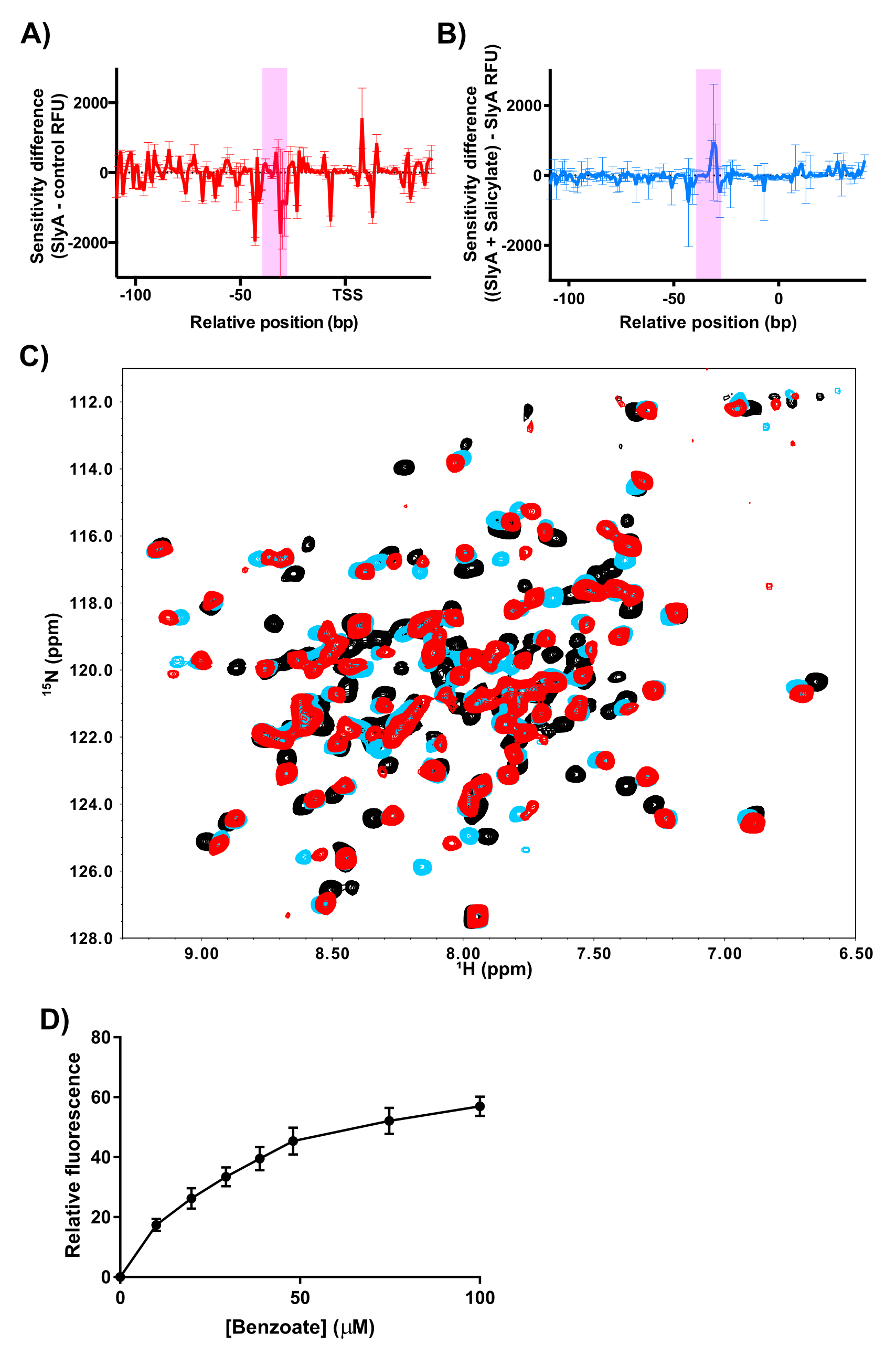

Supplement: FIG S1 [file mBio.00009-19-sf001.tif]
