# Supplementary material for: The Evolution of SlyA/RovA Transcription Factors from Repressors to Countersilencers in Enterobacteriaceae
Source: mBio. 2019 Mar 5;10(2):e00009-19. doi: 10.1128/mBio.00009-19 (PMC6401476; doi:10.1128/mBio.00009-19)
Supplement: TABLE S1 [file mBio.00009-19-st001.docx]

**Table S1. Crystal Structure Statistics.**

Table S1A. Data Collection Statistics.

| Space group | P2_1_2_1_2 |
| --- | --- |
| Unit cell dimensions (a,b,c) (Å) | 63.38 78.02 84.39 |
| Molecules per asymmetric unit | 2 |
| Resolution (Ǻ) (last shell) | 50.0-2.00 Å (2.07-2.00) |
| Unique reflections (last shell) | 27998 (2583) |
| Completeness (last shell) | 96.4% (90.8) |
| Redundancy (last shell) | 12.9 (7.1) |
| <I>/<σ(I)> (last shell) | 31.7 (1.1) |
| R_merge_ (last shell) | 0.074 (>1.00) |

Table S1B. Refinement Statistics.

| Resolution | 20.0 – 2.3 |
| --- | --- |
| R factor (working set) | 0.228 |
| Rfree (test set=5% of the overall) | 0.259 |
| # unique reflections | 17811 |
| Number of protein atoms | 2110 |
| Number of solvent atoms | 19 |
| Number of heteroatoms | 60 (6 salicylates) |
| Wilson B value | 51.8 Å^2^ |
| Average B value from refinement | 64.7 Å^2^ |
| Ramachandran quality | 97.4% in most-favored regions  2.2% in additional allowed regions  0.4% in generously allowed regions |
| rms deviation - bond lengths | 0.011 Å |
| rms deviation - bond angles | 2.0 º |
