# Supplementary material for: The Evolution of SlyA/RovA Transcription Factors from Repressors to Countersilencers in Enterobacteriaceae
Source: mBio. 2019 Mar 5;10(2):e00009-19. doi: 10.1128/mBio.00009-19 (PMC6401476; doi:10.1128/mBio.00009-19)
Supplement: TABLE S2 [file mBio.00009-19-st002.docx]

**Table S2.** **Polymorphisms in salicylate binding sites I and II of SlyA lineage TFs.**

| Site I | T32 | V35 | T36 | I56 | I58 | S62 | T66 |
| --- | --- | --- | --- | --- | --- | --- | --- |
| *Budvicia aquatica* | I |  |  |  |  |  |  |
| *Gibbsiella quercinecans* | I |  |  |  |  |  |  |
| *Phaseolibacter flectens* | I |  |  |  |  |  |  |
| *Pragia fontium* | I |  |  |  |  |  |  |
| *Sodalis glossinidius* | I |  |  |  |  |  |  |
| *Wigglesworthia glossinidia* | I |  |  |  |  |  |  |

| Site II | G6’ | S7’ | A10’ | R14’ | R17’ | W16 | I20 | W34 | V35 | H38 |
| --- | --- | --- | --- | --- | --- | --- | --- | --- | --- | --- |
| *Arsenophonus sp.* |  | T |  |  |  |  |  |  |  | Y |
| *Budvicia aquatica* |  | A |  |  |  |  |  |  | I | Y |
| *Cosenzaea myxofaciens* |  | A |  |  |  |  |  |  |  | Y |
| *Cronobacter sakazakii* |  |  | T |  |  |  |  |  |  |  |
| *Edwardsiella tarda* |  |  | S |  |  |  |  |  |  | Y |
| *Enterobacillus trilobii* |  |  |  |  |  |  |  |  |  | Y |
| *Erwinia gerundensis* |  | T |  |  |  |  |  |  |  |  |
| *Franconia pulveris* |  |  |  |  |  |  |  |  | I |  |
| *Hafnia alvei* |  |  |  |  |  |  |  |  |  | Y |
| *Izhakiella australiensis* |  | T | S |  |  |  |  |  |  |  |
| *Leminorella grimontii* |  | A |  |  |  |  |  |  |  | Y |
| *Moellerella wisconsensis* |  | T |  |  |  |  |  |  |  |  |
| *Obesumbacterium proteus* |  |  |  |  |  |  |  |  |  | Y |
| *Pantoea agglomerans* |  | T | S |  |  |  |  |  | I |  |
| *Pectobacterium carotovorum* |  |  |  |  |  |  | V |  |  |  |
| *Phaseolibacter flectens* | S | I |  |  |  |  |  |  |  |  |
| *Photorhabdus luminescens* |  |  |  |  |  |  |  |  |  | Y |
| *Pragia fontium* |  | A |  |  |  |  |  |  | I |  |
| *Proteus mirabilis* |  | A |  |  |  |  |  |  |  | Y |
| *Providencia rettgeri* |  | T | S |  |  |  |  |  |  | Y |
| *Rosenbergiella nectarea* |  | T |  |  |  |  |  |  |  |  |
| *Shimwellia blattae* |  |  | S |  |  |  |  |  |  |  |
| *Sodalis glossinidius* |  |  |  |  |  |  |  |  | I |  |
| *Tatumella ptyseos* |  | T |  |  |  |  |  |  |  |  |
| *Trabulsiella guamensis* |  |  |  |  |  |  |  |  | I |  |
| *Wigglesworthia glossinidia* |  |  | S |  |  |  |  |  |  |  |
| *Xenorhabdus ishibashii* |  |  |  |  |  |  |  |  |  | Y |
| *Yersinia pseudotuberculosis* |  |  |  |  |  |  |  |  |  | Y |
