# Supplementary material for: The Evolution of SlyA/RovA Transcription Factors from Repressors to Countersilencers in Enterobacteriaceae
Source: mBio. 2019 Mar 5;10(2):e00009-19. doi: 10.1128/mBio.00009-19 (PMC6401476; doi:10.1128/mBio.00009-19)
Supplement: TABLE S3 [file mBio.00009-19-st003.docx]

**Table S3. Oligonucleotides and plasmids used in this study.**

Table S3A. Oligonucleotides.

| Name | Sequence (5’-3’) |
| --- | --- |
| 6-FAM-ydhI-R | 56-FAM/ATTGAGCAGCCATCGCTGG |
| 6HisRovA-F | CTACCATGGAACATCACCATCACCATCACGGTGCTGGAGCATTGGAATCGACATTAGG |
| 6HisRovA-R | CTAGGATCCTTACTTAGTTTGTAATTGAATAATATTTTTC |
| A10P-F | CTAGGTTCTGATCTGCCGCGGTTGGTGCGCATTTGG |
| A10P-R | CCAAATGCGCACCAACCGCGGCAGATCAGAACCTAG |
| BamHI-inv-F | TGCGGATCCTTGGGTAGCGGATAATATTG |
| BamHI-rovA-F | TGCGGATCCGTTGCTGACGAGATG |
| C81A-F | GGCTAATTTCGCGGCAAACCGGCGCCAGCGATCGTCGCGCTAAG |
| C81A-R | CTTAGCGCGACGATCGCTGGCGGAGGTTTGCCGCGAAATTAGCC |
| EcoRI-hnsT-F | AGTCGAATTCAAGGAGCAAAAAAATGATTGATGAATTTC |
| EcoRI-inv-R | TGCAGAATTCGCCGTTGCCCCTCC |
| EcoRI-rovA-F | TACGGAATTCAAGGAGGAGCAATTGGAATCGACATTAGGATCTG |
| EcoRI-rovA-R | TGCAGAATTCGCCATTGGAACAATCTTG |
| EcoRI-slyA-F | TACGGAATTCAAGGAGGAGCAATTGGAATCGCCACTAGGTTCTG |
| G6A-F | GAAATTGGAATCGCCACTAGCTTCTGATCTGGCACGGTTGG |
| G6A-R | CCAACCGTGCCAGATCAGAAGCTAGTGGCGATTCCAATTTC |
| H38A-F | ACACATTGGGTCACGTTGGCGAATATTCATCAATTGCCG |
| H38A-R | CGGCAATTGATGAATATTCGCCAACGTGACCCAATGTGT |
| HindIII-hnsT-R | GCATAAGCTTCAGTCAATGAGATCTTCTGGCG |
| JKP736 | gaccacacccgtcctgtgTGAATGGCTATCTACCAGGG |
| JKP737 | CGCTGGCGCTGGTTTGCCGCGAAATTAGCC |
| JKP738 | CGGCAAACCAGCGCCAGCGATCGTCGCG |
| JKP739 | gatgcgtccggcgtagagGCCGGCCTAACTGGGTAT |
| KMp177 | CGGATCCCCAGATAACGAACCCAAGCG |
| KMp178 | CGGTCCCCAGATGACGAATCCAAACG |
| KMp181 | CAAGCTTTGGTCACATGGCCACACGTAT |
| KMp206 | TGGCGATTCCAATTTCATCTCCTTATAATTAGCTTGCTAAG |
| KMp207 | CTTAGCAAGCTAATTATAAGGAGATGAAATTGGAATCGCCA |
| KpnI-rovA-R | GAGGTACCTTACTTAGTTTGTAATTGAATAATATTTTTCTC |
| KpnI-slyA-R | GAGGTACCTCAATCGTGAGAGTGCAATTCCATAATATTGTGTTC |
| pagC-3'-F | AGAACATTCCACTCAGGATGGCGA |
| pagC-3'-P | TGTAGAGGAGATGTTGCTTCC |
| pagC-3'-R | GACGACGATATTCTCCAGCGGATT |
| rpoAYS-F | CCAAGGTGACCCTTGAGCC |
| rpoAYS-R | TAGTACACCATCAATCTCAACCTCGG |
| R14A-F | CTGGCACGGTTGGTGCAATTGGCGTGCTCTG |
| R14A-R | CAGAGCACGCCAAATTGCCACCAACCGTGCCAG |
| slyA-F | TTGGCTGGGATTTCTTCAGAG |
| slyA-R | TCGTGAGAGTGCAATTCCATAA |
| slyAcomp-F | GGTGCCAAGTGCGCACTATCTCTG |
| slyAcomp-R | GTATGCCCTGCACCTCAATCGTG |
| S7A-F | GGCATTGAGCAGCCAGCGCTGGTACGCACGTTGGATC |
| S7A-R | GATCCAACGTGCGTACCAGCGCTGGCTGCTCAATGCC |
| SlyAreg-F | CGCGGGATCCTTACCGCTGTCCAATGGCTAC |
| SlyAreg-R | CGCGAAGCTTTTCCGACTTCGTTTAAGATTGG |
| STM-Eco-slyA-F | CAGCATAATAACTTAGCAAGCTAATTATAAGGAGATGAAA TTGGAATCGCCACTAGGTTC |
| STM-Eco-slyA-R | CTTTACGTGTGGTCACATGGCCACACGTATGCCCCTGCACCTCACCCTTTGGCCTGTAA |
| STM-slyA-targ-F | ATCAGCATAATAACTTAGCAAGCTAATTATAAGGAGATGAAATAGACAGCTGCATGCAT |
| STM-slyA-targ-R | CTTTACGTGTGGTCACATGGCCACACGTATGCCCCTGCACCTCAGTGTAGGCTGGAGCTG |
| T66A-F | CAGCCATCGCTGGTACGCGCGTTGGATCAACTTGAAGATAAG |
| T66A-R | CTTATCTTCAAGTTGATCCAACGCGCGTACCAGCGATGGCTG |
| W34A-F | TTGACGCAGACACATGCGGTCACGTTGCACAATATTC |
| W34A-R | GAATATTGTGCAACGTGACCGCATGTGTCTGCGTCAA |
| WNp318 | TTGTGAAGTGTATACTCAACGCCACAGGATTGCCCTTACACATATGAATATCCTCCTTAG |
| WNp319 | GTCGCAGATACGCTGTAGTTCCTGTAGCGTGACGGCAAGCGTGTAGGCTGGAGCTGCTTC |
| ydhI-F | GTAAAGAGGGAGAGATCCATTAACA |
| ydhI-R | TGCGCTTGGGTTCGTTAT |

Table S3B. Plasmids.

| Name | Description | Source |
| --- | --- | --- |
| pBAD18 | Arabinose-inducible vector | (69) |
| pET16b::*slyA* | pET16b::6×His-*slyA* | (21) |
| pJK723 | Suicide construct containing C81S mutation | This study |
| pKM05 | 14028s P*_slyA_*-14028s *slyA* ORF chimera | This study |
| pKM07 | *E. coli* K-12 P*_slyA_*-14028s *slyA* ORF chimera | This study |
| pRDH10 | Suicide vector | (63) |
| pRW6 | *pagC* IVT target | (17) |
| pRW20 | IVT scaffold vector | (17) |
| pRW39 | *slyA*/*ydhI* region IVT target | This study |
| pRW54 | *rovA* region IVT target | This study |
| pRW55 | *inv* region IVT target | This study |
| pRW57 | pBAD18::*hnsT*_EPEC_ | This study |
| pRW58 | pBAD18::*slyA* | This study |
| pRW59 | pBAD18::*rovA* | This study |
| pRW60 | pTRC99::6×His-*rovA* | This study |
| pSL2143 | pWSK29 *slyA* | This study |
| pSL2143-G6A | pWSK29 *slyA* G6A | This study |
| pSL2143-S7A | pWSK29 *slyA* S7A | This study |
| pSL2143-A10P | pWSK29 *slyA* A10P | This study |
| pSL2143-R14A | pWSK29 *slyA* R14A | This study |
| pSL2143-H38A | pWSK29 *slyA* H38A | This study |
| pSL2143-T66A | pWSK29 *slyA* T66A | This study |
| pSL2143-C81A | pWSK29 *slyA* C81A | This study |
| pSR47s | Suicide vector | (67) |
| pSW172 | Temperature sensitive plasmid | (65) |
| pTH19Kr | Low copy number vector | (71) |
| pTRC99a | Tightly controlled expression vector | (70) |
| pWSK29 | Low copy number vector | (68) |
