# Supplementary material for: The Evolution of SlyA/RovA Transcription Factors from Repressors to Countersilencers in Enterobacteriaceae
Source: mBio. 2019 Mar 5;10(2):e00009-19. doi: 10.1128/mBio.00009-19 (PMC6401476; doi:10.1128/mBio.00009-19)
Supplement: TABLE S4 [file mBio.00009-19-st004.docx]

**Table S4. Components of IVT reactions.**

| Target promoter | Template | Probe | Primers |
| --- | --- | --- | --- |
| *pagC* | pRW6 | pagC-3’-P | pagC-3’-F/pagC-3’-R |
| *slyA* | pRW39 | slyA-R | slyA-F/slyA-R |
| *ydhI* | pRW39 | ydhI-R | ydhI-F/ydhI-R |
| *inv* | pRW55 | inv-R | inv-F/inv-R |
| *rovA* | pRW54 | rovA-R | rovA-F/rovA-R |
